# Hepatitis viruses infection and risk of intrahepatic cholangiocarcinoma: evidence from a meta-analysis

**DOI:** 10.1186/1471-2407-12-289

**Published:** 2012-07-16

**Authors:** Yanming Zhou, Yanfang Zhao, Bin Li, Jiyi Huang, Lupeng Wu, Donghui Xu, Jiamei Yang, Jia He

**Affiliations:** 1 Department of Hepato-Biliary-Pancreato-Vascular Surgery, First affiliated Hospital of Xiamen University, Xiamen, China; 2 Department of Health Statistics, Second Military Medical University, Shanghai, China; 3 Department of Special Treatment, Eastern Hepatobiliary Surgery Hospital, Second Military Medical University, Shanghai, China

## Abstract

**Background:**

Studies investigating the association between Hepatitis B virus (HBV) and hepatitis C virus (HCV) infections and intrahepatic cholangiocarcinoma (ICC) have reported inconsistent findings. We conducted a meta-analysis of epidemiological studies to explore this relationship.

**Methods:**

A comprehensive search was conducted to identify the eligible studies of hepatitis infections and ICC risk up to September 2011. Summary odds ratios (OR) with their 95% confidence intervals (95% CI) were calculated with random-effects models using Review Manager version 5.0.

**Results:**

Thirteen case–control studies and 3 cohort studies were included in the final analysis. The combined risk estimate of all studies showed statistically significant increased risk of ICC incidence with HBV and HCV infection (OR = 3.17, 95% CI, 1.88-5.34, and OR = 3.42, 95% CI, 1.96-5.99, respectively). For case–control studies alone, the combined OR of infection with HBV and HCV were 2.86 (95% CI, 1.60-5.11) and 3.63 (95% CI, 1.86-7.05), respectively, and for cohort studies alone, the OR of HBV and HCV infection were 5.39 (95% CI, 2.34-12.44) and 2.60 (95% CI, 1.36-4.97), respectively.

**Conclusions:**

This study suggests that both HBV and HCV infection are associated with an increased risk of ICC.

## Background

Intrahepatic cholangiocarcinoma (ICC), which originates from intrahepatic bile ducts, is the second commonest primary hepatic tumour behind hepatocellular carcinoma (HCC), accounting for 3% of all gastrointestinal cancers worldwide [1]. The etiology of ICC is poorly understood although several etiological factors, including hepatolithiasis [2], primary sclerosing cholangitis [3], liver flukes (*Clonorchis sinensis* and *Opisthorchis viverrini)*[4] and exposure to the radiopaque medium thorium dioxide (Thorotrast) [5], has been well established.

It has been shown that Hepatitis B virus (HBV) and hepatitis C virus (HCV) infections are the major causative agent for HCC [6]. Several recent studies have been conducted to investigate the association of ICC with viral hepatitis infections, but the conclusions remained controversial [7-15]. For example, a case–control study from Korea found neither HBV nor HCV infection was associated with the risk of ICC [7]. In contrast, a case–control study from Italy found that seropositivities for anti-HCV and hepatitis B surface antigen (HBsAg) were 25% and 13% in ICC cases and 5.8% and 6.7% in controls, respectively. A statistically significant increase in the odds ratios (OR) was observed for anti-HCV (OR = 9.7), whereas no significant association was found with HBsAg (OR = 2.7) [8]. On the other hand, the results of a case–control study from China showed a positive association of ICC with HBV but not with HCV [9]. Therefore, we conducted a meta-analysis to address the inconsistent results reported in previous studies, thereby improving the estimate of ICC risk in HBV- or HCV- infected patients.

## Methods

### Study selection

To identify the relevant literature, searches of PubMed, Embase, Ovid, Cochrane Library, and Scopus database for articles on ICC associated with HBV or HCV infection were conducted up to September 2011. The following MeSH (Medical Subject Heading) search headings were used: ‘hepatitis B virus’,” “hepatitis C virus,” “bile duct neoplasms”, and “cholangiocarcinoma”. Reference lists of all retrieved articles were manually searched for additional studies. Serum HBsAg and hepatitis C antibody were used as the positive markers of chronic hepatitis virus infection.

### Criteria for inclusion and exclusion

The inclusion criteria in the meta-analysis are as follows: published full-text report in English language, studies provided sufficient data to calculate the risk estimates with its corresponding 95% confidence interval (CI) of ICC associated with HBV or HCV infection.

Abstracts, letters, editorials and expert opinions, reviews without original data, case reports and studies lacking control groups were excluded. The following studies were also excluded: 1) those evaluating patients with HCC or liver metastase; 2) those with incomplete raw data; 3) those with repetitive data.

### Data extraction

Two reviewers (B.L. and Y.Z.) independently extracted the following parameters from published studies: the name of the first author, publication year, study design, the country in which the study was conducted, sample size, prevalence of HBV or HCV seropositivity in cases and in a control group or in cohort, and OR or hazard ratios (HR) estimates with 95% CI for HBV or HCV infection and ICC.

### Quality assessment

The methodological quality of the included studies assessed using a three-items scoring system measured by the study design (cohort =1; case–control =0), sample size (>100 =1; 100 = 0), and reported outcomes of interest (both HBV and HCV =1; HBV or HCV only = 0). Studies having a score of 2 were considered to be of high quality.

### Statistical methods

The literature review refered to PRISMA statement standards.We extracted adjusted OR and HR with 95% CI from the included studies. Summary OR was estimated using random-effects models. Heterogeneity was calculated by means of Q test and *χ*2 test. Publication bias was assessed visually using a funnel plot. All analyses were conducted using Review Manager (RevMan) software 5.0.

## Results

### Selection of studies

**Figure 1:**
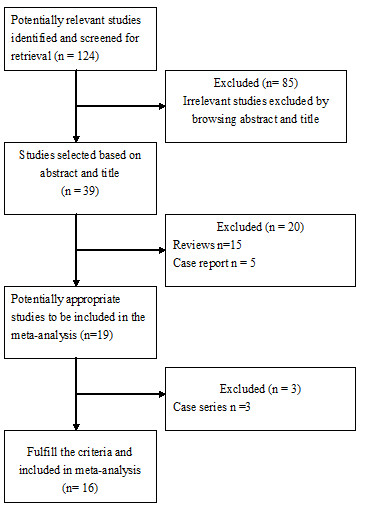
Literature flow chart.

Figure 1 shows the flow chart of publications identified by the literature search. A total of 13 case–control studies [7-19] and 3 cohort studies [20-22] were included in the meta-analysis. The main features of the studies included in the meta-analysis are shown in Tables 1 and 2. Among the 13 case–control studies, three were from United States [10,11,15], three from China [9,18,19], three from Korea [7,14,16], one from Italy [8], one from Thailand [13], one from Taiwan [17], and one from Japan [12]. Control subjects originated from hospital-based [7-9,11-14,16-19], or general population-based population [10,15].

**Table 1:** Characteristics of case–control studies of hepatitis viruses infection and ICC risk

**First author**	**Year**	**Country**	**Cases** (***n***)	**Control** (***n***)	**Control description**	**OR (95% CI) ** **for HBV infection**	**OR (95% CI) ** **for HCV infection**	**Study quality**
Parkin	1991	Thailand	100	100	Hospital-based control	1.0 (0.4-2.1)	–	1
Shin	1996	Korea	41	406	Hospital-based control	1.3 (0.3-5.3)	3.9 (0.9-17.1)	2
Donato	2001	Italy	26	824	Hospital-based control	2.7 (0.4-18.5)	9.7 (1.6-58.9)	2
Yamamoto	2004	Japan	50	205	Hospital-based control	1.8 (0.3-10.1)	16.8 (5.7-50.0)	2
Shaib	2005	US	625	90834	Population-based control	0.8 (0.1-5.9)	6.1 (4.3-8.6)	2
Choi	2006	Korea	185	185	Hospital-based control	0.8 (0.198-3.023)	1.0(0.04-25.264)	2
Shaib	2007	US	83	236	Hospital-based control	2.9 (0.1-236.8)	7.9 (1.3-84.5)	2
Welzel	2007	US	535	102782	Population based control	–	4.4 (1.4-14.0)	1
Lee	2008	Korea	622	2488	Hospital-based control	2.3 (1.6-3.3)	1.0 (0.5-1.9)	2
Zhou	2008	China	312	438	Hospital-based control	8.876 (5.98-13.19)	0.933 (0.281-3.1)	2
Lee	2009	Taiwan	160	160	Hospital-based control	4.985 (2.78-8.95)	2.71 (1.16-6.32)	2
Tao	2010	China	61	380	Hospital-based control	18.1 (7.5-44.0)	–	1
Peng	2011	China	98	196	Hospital-based control	2.75 (1.27-5.95)	–	1

ICC, intrahepatic cholangiocarcinoma; HBV,hepatitis B virus; HCV, hepatitis C virus; US, United States.

**Table 2:** Characteristics of cohort studies of hepatitis viruses infection and ICC risk

**First author**	**Year**	**Country**	**Source**	**Sample size**	**Mean follow-up** ** (years)**	**ICC**	**Hazard ratio 95% CI**	**Study ,** **quality**
Tanaka	2009	Japan	Voluntary blood donors	2519 (HBsAg +)	7.6	2	8.56 (1.33-55.2)	3
				1927 (anti-HCV+)		1	2.63 (.25-27.73)	
				150368 (all negative)		8		
El-Serag	2009	US	Veterans population	146394 (anti-HCV+)	2.3	14	2.6 (1.3-5.0)	2
				572293 (anti-HCV–)		23		
Fwu	2011	Taiwan	Pregnant women	289992 (HBsAg +)	6.91	9	4.80 (1.88-12.2)	2
				1492409 (HBsAg –)		9		

ICC, intrahepatic cholangiocarcinoma; HBsAg, hepatitis B surface antigen; HCV, hepatitis C virus; US, United States.

Among the 3 cohort studies, one was from United States [20], one from Japan [21], and one from Taiwan [22]. The average observation period ranged from 2.3 to 7.6 years.

The two reviewers had 100% agreement in their reviews of the data extraction.

### Meta-analysis

**Figure 2:**
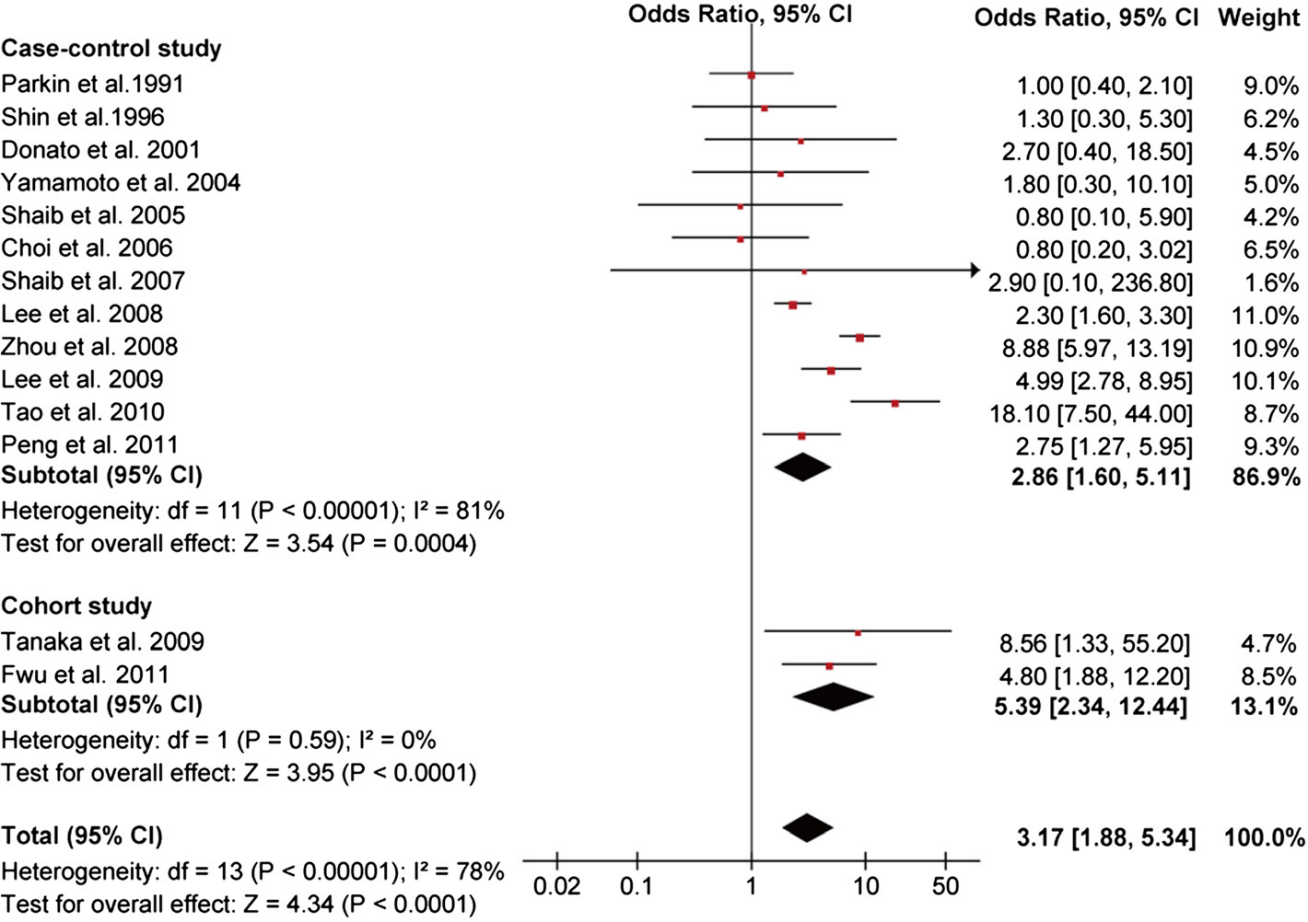
Forest plot of intrahepatic cholangiocarcinoma risk associated with HBV infection.

**Figure 3:**
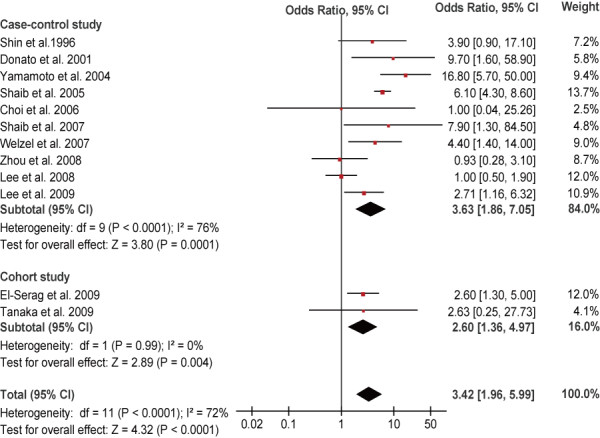
Forest plot of intrahepatic cholangiocarcinoma risk associated with HCV infection.

Meta-analysis of all these 16 studies in a random-effects model found statistically significant increased risk of ICC incidence with HBV and HCV infection (OR = 3.17, 95% CI, 1.88-5.34, *P* < 0.001 and OR = 3.42, 95% CI,1.96-5.99, *P* < 0.001, respectively). When case–control studies were analyzed alone, the combined OR for the association of HBV and HCV infection with the risk for ICC were 2.86 (95% CI, 1.60-5.11) and 3.63 (95% CI, 1.86-7.05), respectively. When cohort studies were analyzed alone, the combined OR of HBV and HCV infection were 5.39 (95% CI, 2.34-12.44) and 2.60 (95% CI, 1.36-4.97), respectively. All these results were significant heterogeneous (*P* < 0.001) (Figures 2 and 3).

#### Sensitivity analysis of high quality studies

Analysis of these studies [7-12,14,16,17,20-22] revealed both HBV (OR = 3.10, 95% CI, 1.77-5.42, *P* < 0.001) and HCV infection (OR = 3.35, 95% CI, 1.82-6.15, *P* < 0.001) were associated with significant increased risk of ICC development.

### Publication bias

**Figure 4:**
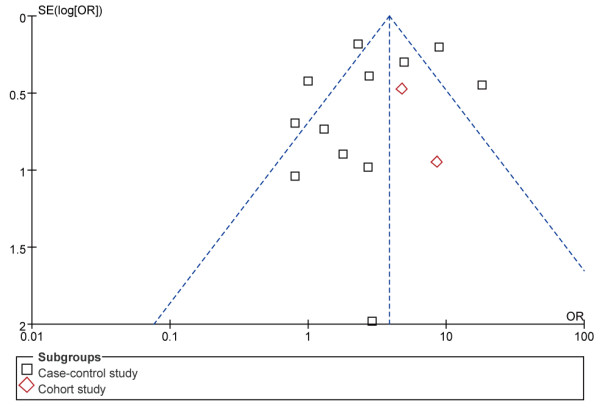
Funnel plot of studies evaluating the association between HBV infection and intrahepatic cholangiocarcinoma risk.

A “funnel plot” of the studies in the meta-analysis reporting HBV infection and ICC is shown in Figure 4. Five of the studies lay outside the 95% confidence interval boundaries, and significant heterogeneity was observed.

## Discussion

Meta-analysis was employed by a recently published study to estimate the correlation between hepatitis virus infection and the risk of ICC and extrahepatic cholangiocarcinoma (ECC) [23]. Because different anatomic location of CC has distinct epidemiologic features indicating different sets of risk factors [11], here we used meta-analysis to examine the relationship between HBV/HCV infection and the risks of ICC, and restricted our analysis to patients only with ICC.

In this meta-analysis, we found positive relationship between HBV/HCV infection and the development of ICC. This conclusion is further supported by the evidences from a series of experimental studies. In a study from the United States, HBV and/or HCV DNA was present in 3 (27%) out of 11 ICC tissue samples obtained at the time of surgical resection [24]. Another study from China also showed that HBV DNA was detected in 34.8% (8/23) of ICC cases [25]. Recently, Torbenson *et al.*[26] found that dysplasia of the intrahepatic bile ducts, the histologic precursor lesion of ICC, was present in HCV related cirrhotic livers.

The mechanism of the development of ICC in patients with HBV or HCV infection is still uncertain. HBV and HCV infection can cause chronic inflammation of the liver. Indeed, chronic inflammation and cancer are closely associated. In this context, long-term expression of several viral oncoproteins, mostly the HCV core protein and HBx protein, might participate in the tumourigenic process. Battaglia *et al.*[27] found that HCC derived-HCV core variants alleviate TGF-beta cytostatic responses and increase TGF-beta-mediated epithelial to mesenchymal transition (EMT) in mouse or human primary hepatocytes. Interestingly, Li *et al.*[28] reported that HCV core protein expression can induce EMT in cholangiocarcinoma cell line. On the other hand, HBx acting as a transactivator on various cellular genes that are involved in the control of the cell cycle, proliferation or apoptosis has been shown to be present in some HBV-infected ICC specimens [29]. There are reports providing evidence that HBx gene transfection can upregulate the transcriptional expression of human telomerase reverse transcriptase mRNA both in HCC and cholangiocarcinoma cell lines [30]. Clinically, Peng *et al.*[19] reported that ICC patients with HBV infection were more common in male and younger patients as compared to ICC patients with seronegative HBsAg, similar to HCC patients. Furthermore, the profile of age distribution of ICC patients with HBV infection was roughly close to that of HCC patients. Similarly, Lee *et al.*[17] also found that both viral hepatitis-associated ICC and HCC had similar age profiles and difference in age distributions between patients with chronic hepatitis B and patients with chronic hepatitis C. In addition, the surgical outcomes of ICC patients with hepatitis, preoperatively diagnosed as HCC, is favorable, similar to that of typical HCC patients [31]. Taking these clinical and experimental findings into account, it seems to be reasonable that hepatitis virus–associated ICC and HCC share common disease processes for carcinogenesis.

Some researchers suggest that hepatocytes and cholangiocytes might originate from hepatic progenitor cells (HPCs) [32]. Therefore it is possible that both ICC and HCC in patients infected with HBV and HCV may be derived from HPCs. In fact, cumulative studies have provided strong evidence to this hypothesis. Cumulative evidence suggests that HBV- and HCV-associated HCC and ICC are derived from HPCs. (i) It has been demonstrated that HPCs can be infected with HBV and HCV, and that proliferation of large numbers of HPCs was seen in HBV- and HCV-associated cirrhosis which is a risk factor for liver cancer [33,34]. (ii) Based on gross morphological features, ICC are classifiable into three representative types of growth patterns: the mass-forming (MF) type, the periductal infiltrating (PI) type, and the intraductal growth (IG) type [35]. It is likely that PI and IG type tumors arise from malignant transformation of epithelial cells lining the larger bile ducts, whereas the MF type arises from smaller bile ducts or liver progenitor cells within portal areas [36]. Yamamoto *et al.*[37] speculated that HCV-infected proliferating cholangioles in patients with chronic hepatitis C might be associated with the development of MF type ICC. Yu *et al.*[36] found that viral hepatitis-associated ICC was more likely to be of the MF type rather than the PI and IG type. (iii) Alpha-fetoprotein (AFP) is a marker of HPCs compartments [38]. Viral hepatitis-associated ICC patients were found had elevated serum alpha-fetoprotein levels as compared with seronegative ICC patients [19,36]. One possible mechanism for the development of ICC is that the neoplastic transformation of HPCs is involved in the genesis of ICC and that the HPCs retain their ability to produce AFP through the process of malignant transformation [38]. Indeed, Ishii *et al.*[39] demonstrated that AFP-producing cells in cholangiocarcinomas possessed cancer stem cell-like properties. More importantly, one most recent study from China found that HBx induces intrinsic cellular transformation, promoting the expansion and tumorigenicity of HPCs in 3,5-diethoxycarbonyl- 1, 4-dihydrocollidine treated mice [40].

ICC is characterized by wide variability in incidence and risk factors. HCV seems to be associated with ICC in regions with relatively low prevalence of HBV infection such as United States, Italy and Japan. In contrast, several studies from China [9,18,19], a highly endemic area for HBV infection, found that not HCV but HBV infection was significantly associated with ICC. On the other hand, a recent study from Taiwan, where both HBV and ICC are endemic, found that both HBV and HCV are significantly associated with ICC [17]. These studies indicate that the different endemic hepatitis virus types in different regions may be a determinant to the variability of risk factors for ICC.

Our study also has some weaknesses which should be considered when interpreting results. First, seropositivity for HBsAg and anti-HCV was used as the sole indicator of HBV and HCV infection. It seems that occult HBV and HCV infection may also play a role in the development of HCC [41]. Therefore, the definition in the present report might result in underestimation of the effect of hepatitis viruses infection. Second, there were significant heterogeneities between different studies, which would lower the reliability of the summary odds ratio. The presence of heterogeneity is most likely to have been introduced by diversity in study design, population demographics, location of studies conducted and adjustments for confounders. Finally, the possibility of publication bias is of concern. The fact that studies with positive results are more likely to be published than negative and non-English studies were excluded in the current meta-analysis might be sources of potential publication bias.

## Conclusions

In conclusion, in this meta-analysis of 13 case–control studies and 3 cohorts, we found that HBV and HCV infection are associated with an increased risk of ICC.

## Misc

Yanming Zhou, Yanfang Zhao contributed equally.

## Competing interests

The authors declare that they have no competing interests.

## Authors’ contributions

YZ participated in the design and coordination of the study, carried out the critical appraisal of studies and wrote the manuscript. BL, LW, JH, and DX developed the literature search, carried out the extraction of data, assisted in the critical appraisal of included studies and assisted in writing up. YZ and JH carried out the statistical analysis of studies. YZ and JY interpreted data, corrected and approve the manuscript. All authors read and approved the final manuscript.

## Pre-publication history

The pre-publication history for this paper can be accessed here:

http://www.biomedcentral.com/1471-2407/12/289/prepub
